# Ablation of the gut microbiota alleviates high-methionine diet-induced hyperhomocysteinemia and glucose intolerance in mice

**DOI:** 10.1038/s41538-023-00212-3

**Published:** 2023-07-17

**Authors:** Wenqiang Li, Yiting Jia, Ze Gong, Zhao Dong, Fang Yu, Yi Fu, Changtao Jiang, Wei Kong

**Affiliations:** 1 Department of Physiology and Pathophysiology, School of Basic Medical Sciences, Peking University Health Science Center, Beijing, China; 2 State Key Laboratory of Vascular Homeostasis and Remodeling, Peking University, Beijing, China; 3 Center of Basic Medical Research, Institute of Medical Innovation and Research, Third Hospital, Peking University, Beijing, China; 4 Center for Obesity and Metabolic Disease Research, School of Basic Medical Sciences, Peking University, Beijing, China

**Keywords:** Metagenomics, Policy and public health in microbiology

## Abstract

A high-methionine (HM) diet leads to hyperhomocysteinemia (HHcy), while gastrointestinal tissue is an important site of net homocysteine (Hcy) production. However, the role of the gut microbiota in host HHcy remains obscure. This study aimed to determine whether gut microbiota ablation could alleviate host HHcy and glucose intolerance and reveal the underlying mechanism. The results showed that the HM diet-induced HHcy and glucose intolerance in mice, while antibiotic administration decreased the plasma level of Hcy and reversed glucose intolerance. HM diet increased intestinal epithelial homocysteine levels, while antibiotic treatment decreased intestinal epithelial homocysteine levels under the HM diet. Gut microbiota depletion had no effect on the gene expression and enzyme activity of CBS and BHMT in the livers of HM diet-fed mice. The HM diet altered the composition of the gut microbiota with marked increases in the abundances of *Faecalibaculum* and *Dubosiella*, which were also positively correlated with plasma Hcy concentrations. An in-depth analysis of the bacterial cysteine and methionine metabolism pathways showed that the abundances of two homocysteine biosynthesis-related KEGG orthologies (KOs) were markedly increased in the gut microbiota in HM diet-fed mice. Hcy was detected from *Dubosiella newyorkensis*-cultured supernatant by liquid chromatography–tandem mass spectrometry (LC‒MS) analysis. In conclusion, these findings suggested that the HM diet-induced HHcy and glucose intolerance in mice, by reshaping the composition of the gut microbiota, which might produce and secrete Hcy.

## Introduction

Hyperhomocysteinemia (HHcy) is defined as an elevated level of plasma homocysteine (>15 μmol/L)^1,2^, which is a sulfur-containing amino acid produced during methionine metabolism^3^. Numerous clinical studies have shown that HHcy is an independent risk factor for cardiovascular diseases, including atherosclerosis, hypertension, vascular calcification, and aneurysm^4–10^. Previous studies also showed that HHcy promoted glucose intolerance, insulin resistance^11,12^, and hepatic steatosis in mice^13^. Plasma levels of homocysteine (Hcy) are affected by various factors, such as genetic deficiency, high methionine intake, lack of vitamins B6/B12 or folic acid, and administration of certain drugs^14–17^. In humans, a single dose of methionine intake (100 mg/kg body weight) can increase plasma Hcy concentrations^18^. Moreover, a high-methionine (HM) diet has been widely adopted to induce HHcy in experimental animals^19^. Metabolism of excess methionine to homocysteine is a vital biological process composed of a series of reactions catalyzed by metabolic enzymes, including MAT and AHCY, which are abundantly expressed in the liver^20–22^. Therefore, the liver is considered the main site of methionine and homocysteine metabolism^23^. Of interest, recent studies in piglets using the isotope-labeling method showed that 20% of the dietary methionine intake was metabolized by the gastrointestinal tissue (GIT) and was metabolized into homocysteine (31%), tissue protein (29%), or CO_2_ (40%) in the GIT. The GIT contributed to net homocysteine production^24^. However, the role of the GIT in HHcy remains unclear.

The gastrointestinal tract harbors the gut microbiota, which influences host health and disease^25^. Unlike the host genome, the gut microbiome exhibits plasticity and can respond to numerous environmental factors. Of these environmental factors, diet emerges as a significant determinant of gut microbiota composition and function. For example, a high-fat diet caused a decline in the proportion of *Bacteroidetes* and an increase in both *Firmicutes* and *Proteobacteria* in mice^26^. The gut microbiota also plays a crucial role in host metabolism, including amino acid metabolism. For instance, in the gut, the three major tryptophan metabolism pathways producing indole, serotonin, and kynurenine derivatives are directly or indirectly affected by the microbiota^27^. The intestinal contents of germ-free or antibiotic-treated mice were deficient in certain tryptophan metabolites, likely because several intestinal bacteria encode tryptophan metabolic enzymes^28,29^. However, whether the gut microbiota participates in the metabolism of other amino acids is still unclear.

In this study, we revealed that gut microbiota ablation alleviated HM diet-induced hyperhomocysteinemia and glucose intolerance in mice. We proposed that under the HM diet, the composition of the gut microbiota was altered to produce and secrete more homocysteine, which induced host hyperhomocysteinemia and glucose intolerance.

## Results

### Gut microbiota ablation alleviated hyperhomocysteinemia in HM diet-fed mice

**Fig. 1:**
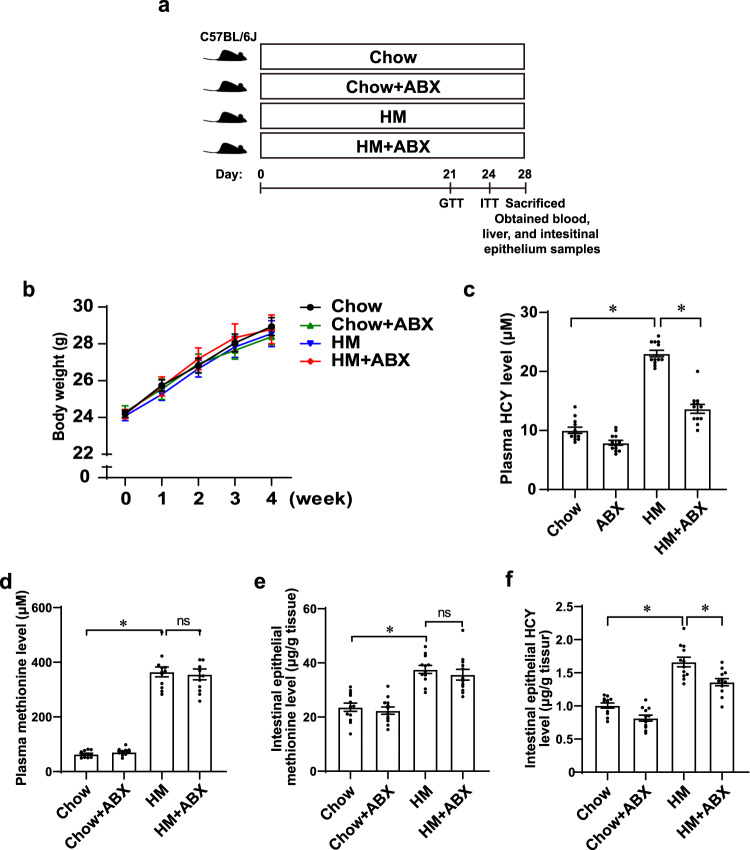
Antibiotic treatment decreased plasma homocysteine levels in HM diet-fed mice. **a** Schematic diagram of the experiment. Body weight **b** was recorded throughout the 4-week experimental period. **c** Plasma homocysteine level. **d** Plasma methionine level. **e** Intestinal epithelial methionine level. **f** Intestinal epithelial homocysteine level. HM high methionine, ABX antibiotics, GTT glucose tolerance test, ITT insulin tolerance test, HCY homocysteine. The values are expressed as the means ± SEM, *n* = 6 per group (**b**), *n* = 12 per group (**c**–**f**). Significance was determined by unpaired two-tailed Student’s *t*-test (**b**) and unpaired one-way ANOVA with Tukey’s post hoc test (**c**–**f**). *P < 0.05, ns: not significant.

Male C57BL/6J mice (8 weeks old) were fed a chow diet or a high-methionine (HM) diet for 4 weeks, which is regarded as a commonly used hyperhomocysteinemia mouse model. To determine the importance of the gut microbiota for HM diet-induced hyperhomocysteinemia, we applied nonabsorbable broad-spectrum antibiotic (ABX) treatment in drinking water to ablate the gut microbiota (Fig. 1a). ABX treatment resulted in a marked reduction in gut microbial abundance (*P* < 0.0001; Supplementary Fig. 1). There were no significant differences in food intake or body weight among the four groups (Supplementary Fig. 2 and Fig. 1b). Plasma homocysteine levels in HM diet-fed mice were significantly elevated compared to those in chow diet-fed mice (23.04 vs. 10.04 μM, *P* < 0.0001; Fig. 1c). Moreover, ABX treatment significantly reduced the plasma level of homocysteine (13.67 vs. 23.04 μM, *P* < 0.0001; Fig. 1c), demonstrating that the gut microbiota is required for HM diet-induced hyperhomocysteinemia. HM diet increased plasma methionine levels compared to chow diet (364.7 vs. 63.6 μM, *P* < 0.0001; Fig. 1d), while there was no significant difference between HM group and HM + ABX group (Fig. 1d). HM diet increased intestinal epithelial methionine levels compared to chow diet (37.58 vs. 23.64 μg/g, *P* < 0.0001; Fig. 1e), while there was no significant difference between HM group and HM + ABX group (Fig. 1e). HM diet increased intestinal epithelial homocysteine levels compared to chow diet (1.664 vs. 1.007 μg/g, *P* < 0.0001; Fig. 1f), and ABX treatment reduced intestinal epithelial homocysteine levels under HM diet (1.360 vs. 1.664 μg/g, *P* = 0.0014; Fig. 1f), implying intestinal epithelium tissue exported less homocysteine with ABX treatment.

### Gut microbiota ablation alleviated glucose intolerance in HM diet-fed mice

**Fig. 2:**
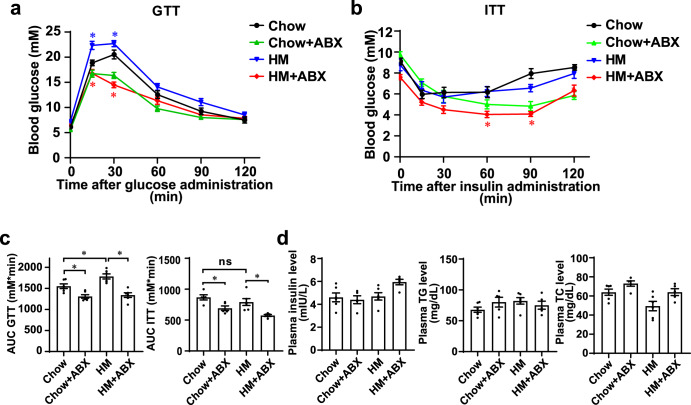
Antibiotic treatment alleviated glucose intolerance in HM diet-fed mice. **a** GTT of the four groups. **b** ITT of the four groups. **c** The associated area under the curve (AUC) values of the GTT and the AUC values of the ITT. **d** Plasma insulin level, plasma TG level, and plasma TC level of the four groups. GTT glucose tolerance test, ITT insulin tolerance test, TG triglyceride, TC total cholesterol. The values are expressed as the means ± SEM, *n* = 6 per group. Significance was determined by unpaired one-way ANOVA with Tukey’s post hoc test. *P < 0.05, ns: not significant.

To explore the effect of the gut microbiota under the HM diet on glucose metabolism, we performed a glucose tolerance test (GTT) and insulin tolerance test (ITT) on the four groups of mice. The GTT revealed marked glucose intolerance and increased the area under the blood glucose curve (*P* = 0.0326) in the HM diet-fed mice, while the HM diet had no apparent effect on insulin resistance (*P* = 0.5915; Fig. 2a–c). ABX treatment significantly improved glucose tolerance (*P* < 0.0001; Fig. 2a and c), indicating that the gut microbiota is required for HM diet-induced glucose intolerance. There were no significant differences in plasma insulin, triglyceride (TG), and total cholesterol (TC) levels between groups (Fig. 2d).

### Antibiotic treatment did not affect homocysteine metabolism in the livers of HM diet-fed mice

**Fig. 3:**
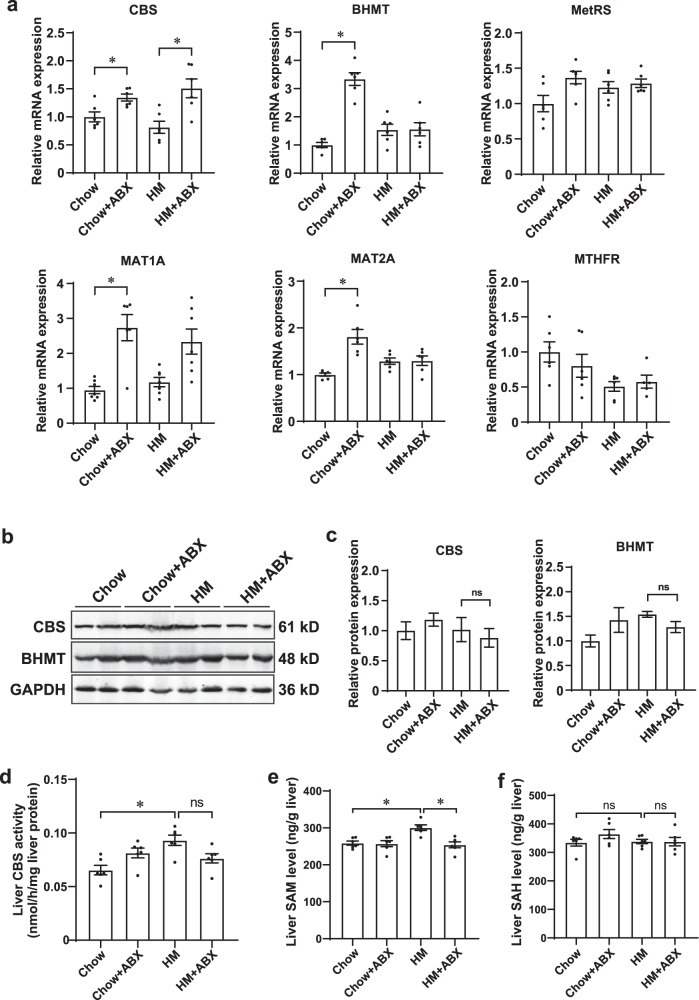
Gut microbiota ablation had no effect on the gene expression and enzyme activity of homocysteine metabolic enzymes in the livers of HM diet-fed mice. **a** mRNA level of CBS, BHMT, MetRS, MAT1A, MAT2A, and MTHFR in the liver tissues of the four groups. **b**, **c** Western blot analysis of CBS and BHMT in the liver tissues of the four groups. **d** Liver CBS enzymatic activity of the four groups. **e** Liver SAM level. **f** Liver SAH level. SAM S-adenosylmethionine, SAH S-adenosyl-l-homocysteine. The values are expressed as the means ± SEM, *n* = 6 per group. Significance was determined by unpaired one-way ANOVA with Tukey’s post hoc test. *P < 0.05, ns: not significant.

The mechanism of the gut microbiota in HM diet-induced hyperhomocysteinemia is still unclear. We first hypothesized that the gut microbiota under an HM diet might change host homocysteine metabolism by generating undiscovered metabolites. Methionine is transmethylated to form homocysteine via S-adenosylmethionine (SAM). Homocysteine is either remethylated to synthesize methionine catalyzed by betaine-homocysteine methyltransferase (BHMT) or undergoes transsulfuration to form cysteine catalyzed by cystathionine β-synthase (CBS). The liver is a major site of methionine and homocysteine metabolism^30^, as BHMT and CBS are predominantly expressed in the liver^31^. We assessed the gene expression levels and enzyme activity of liver homocysteine metabolic enzymes. The HM diet showed no effect on the hepatic mRNA and protein expression levels of CBS, BHMT, and other homocysteine metabolism-related genes, including methionyl-tRNA synthetase (MetRS), methionine adenosyltransferase 1A (MAT1A), methionine adenosyltransferase 2A (MAT2A), and methylenetetrahydrofolate reductase (MTHFR) (Fig. 3a). Antibiotics treatment increased the mRNA expression levels of CBS, BHMT, MAT1A, and MAT2A compared to chow diet group, which was consistent with decreased plasma homocysteine levels between chow + ABX group and chow group (7.92 vs. 10.04 μM, *P* = 0.0566; Fig. 1d). Although HM + ABX upregulated the mRNA abundance of CBS compared to HM (*P* = 0.0017; Fig. 3a), the protein levels of CBS and BHMT remained unchanged in the liver (*P* = 0.9236, *P* = 0.6450; Fig. 3b and c). Moreover, the enzymatic activity of CBS was elevated under the HM diet (*P* = 0.0017), which was consistent with previous studies^32^ (Fig. 3d). However, the enzymatic activity of CBS was not altered upon deletion of the gut microbiota in mice (*P* = 0.0701; Fig. 3d). Taken together, the current data indicated that gut microbiota ablation had no effect on the gene expression levels and enzyme activity of homocysteine metabolic enzymes in the livers of HM diet-fed mice.

We found that HM diet increased liver SAM levels (300.6 vs. 258.8 ng/g, *P* = 0.0033) and HM + ABX decreased liver SAM levels compared to HM (254.1 vs. 300.6 ng/g, *P* = 0.0012; Fig. 3e), implying that ABX treatment alleviated liver methionine overload. There were no significant differences in liver S-adenosyl-l-homocysteine (SAH) levels between groups (Fig. 3f). We also detected intestinal epithelial mRNA expression levels of MetRS, MAT2A, BHMT, and SLC1A5 (solute carrier family 1 member 5). There were no significant differences in the mRNA abundance of homocysteine metabolism-related genes and homocysteine transporters among the four groups (Supplementary Fig. 3). As shown in Supplementary Fig. 4, the HM diet did not affect the normal colonic structure. However, the HM + ABX group showed a disorganized colonic glandular structure, atrophy of the crypt structure, and lymphocyte infiltration compared to the HM group (Supplementary Fig. 4). Whether colonic mucus layer structure alterations are related to decreased plasma homocysteine levels remains unclear.

### The HM diet altered the gut microbiota composition

**Fig. 4:**
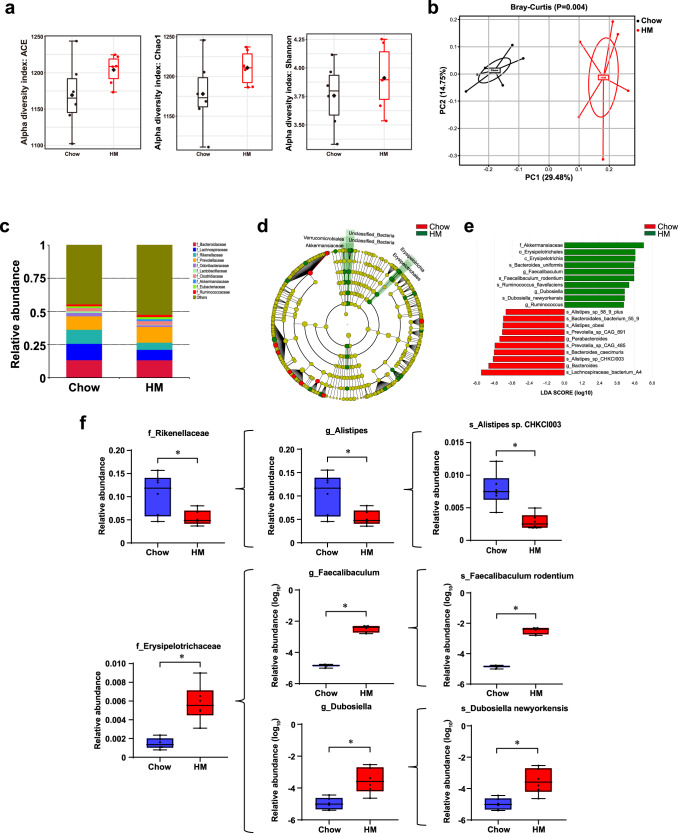
The HM diet altered the gut microbiota structure in mice. **a** α-Diversity of the gut microbiota between chow diet-fed mice and HM diet-fed mice, as indicated by the ACE, Chao1, and Shannon indices. **b** PCoA of the Bray‒Curtis distance matrix. **c** Microbiota composition at the family level. **d**, **e** Taxonomic cladogram generated from LEfSe of metagenomic sequencing data. Green indicates enriched taxa in the HM group. Red indicates enriched taxa in the Chow group. **f** The relative abundances of *f_Rikenellaceae*, *f_Erysipelotrichaceae*, *g_Alistipes*, *g_Faecalibaculum*, *f_Dubosiella*, *s_Faecalibaculum rodentium*, and *s_Dubosiella newyorkensis*. For box plots, the midline represents the median, the box represents the interquartile range (IQR) between the first and third quartiles, and the whiskers represent the lowest or highest values within 1.5 times the IQR from the first or third quartiles. *n* = 6 per group. Significance was determined by the Mann‒Whitney test. *P < 0.05.

To evaluate compositional shifts in the gut microbiota, we performed metagenomic shotgun sequencing of fecal samples obtained from chow diet-fed mice (*n* = 6) and HM diet-fed mice (*n* = 6) on Day 28. As shown by rarefaction curves, these curves became much flatter, indicating that the gene richness approached saturation in each group (Supplementary Fig. 5). There was no significant difference in the α diversity between the two groups, as indicated by the abundance-based coverage estimator (ACE), Chao1, and Shannon indices (Fig. 4a). Ordination of Bray‒Curtis dissimilarity by principal coordinate analysis (PCoA) confirmed the shifts in the gut microbiota composition of the two groups (*P* = 0.004; Fig. 4b). The HM diet-induced difference in the microbiota composition is illustrated in Fig. 4c. Under the HM diet, the bacterial families *Lachnospiraceae* and *Rikenellaceae* were decreased, while *Prevotellaceae* was increased (Fig. 4c). We next performed linear discriminant analysis effect size (LEfSe) analysis and identified several bacterial genera and species that were significantly different between the chow and HM groups. The abundance of *Erysipelotrichales* was higher in the HM group than in the chow group (Fig. 4d and e). HM diet-fed mice had a higher relative abundance of *Erysipelotrichaceae* (*P* = 0.0022) but a lower relative abundance of *Rikenellaceae* (*P* = 0.0411). Additionally, *Faecalibaculum* (*P* = 0.0022) and *Dubosiella* (*P* = 0.0043), two genera in the *Erysipelotrichaceae* family, were enriched, while the *Alistipes* genus (*P* = 0.0411) from the *Rikenellaceae* family was decreased in the HM group. At the species level, HM increased the relative abundances of *Faecalibaculum rodentium* and *Dubosiella newyorkensis* (*P* = 0.0022, *P* = 0.0043; Fig. 4f). Together, these data suggested that the gut microbiota structure was altered by the HM diet, likely contributing to the high level of circulating homocysteine in mice.

### HM diet-induced functional shifts in the gut microbiota increased bacterial homocysteine production

**Fig. 5:**
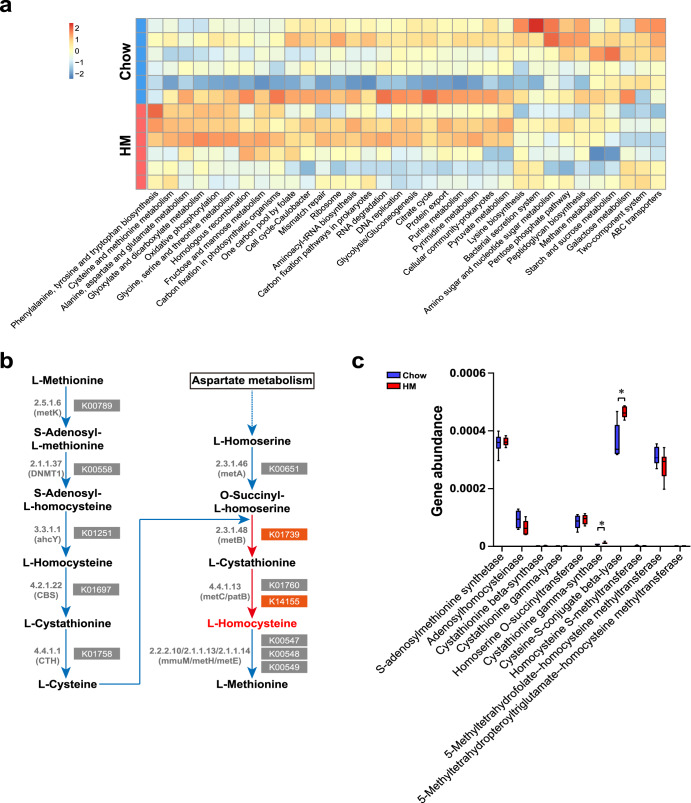
The intestinal bacterial ability to produce homocysteine was enhanced under the HM diet. **a** Hierarchical clustering of the heatmap displaying the significantly changed KEGG pathways. **b**, **c** Bacterial methionine degradation and methionine biosynthesis pathway showing KOs that significantly increased under the HM diet. For box plots, the midline represents the median, the box represents the IQR between the first and third quartiles, and the whiskers represent the lowest or highest values within 1.5 times the IQR from the first or third quartiles. *n* = 6 per group. Significance was determined by the Mann‒Whitney test. *P < 0.05.

To further investigate the functional changes in the gut microbiota in response to the HM diet, we annotated genes to Kyoto Encyclopedia of Genes and Genomes (KEGG) pathways. The cysteine and methionine metabolism pathway was upregulated in the HM group (*P* = 0.0371; Fig. 5a). Cysteine and methionine metabolism include methionine degradation and methionine biosynthesis. The de novo methionine biosynthesis pathway is present across prokaryotes^33^ but is absent from vertebrates, which must obtain methionine through external sources. In-depth analysis of the bacterial cysteine and methionine metabolism pathways showed that two methionine biosynthesis-related KEGG orthologies (K01739 and K14155) were markedly increased in the gut microbiota from HM diet-fed mice (*P* = 0.0235, *P* = 0.0260; Fig. 5b). Cystathionine gamma-synthase (CGS; EC 2.5.1.48) and cysteine-S-conjugate beta-lyase (CCBL; EC 4.4.1.13) were involved in bacterial homocysteine production in the de novo methionine biosynthesis pathway (Fig. 5b and c). Collectively, these results supported the conclusion that the HM diet-induced hyperhomocysteinemia in mice, by increasing intestinal microbial homocysteine production.

### *Faecalibaculum* and *Dubosiella* potentially produced homocysteine

**Fig. 6:**
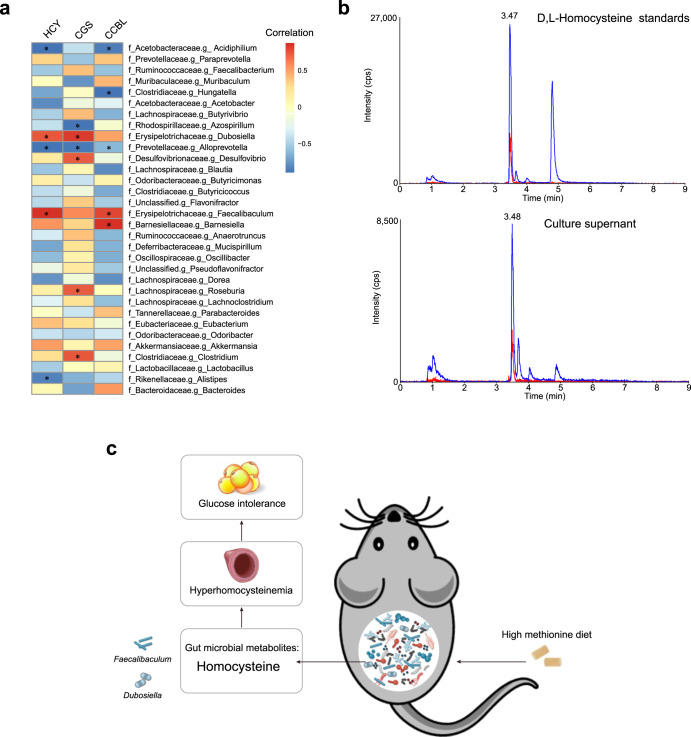
*Faecalibaculum* and *Dubosiella* potentially produced homocysteine. **a** Heatmap of Spearman’s correlation between the abundance of the gut microbiota and the mouse plasma level of homocysteine and the bacterial abundance of CGS and CCBL. **b** LC‒MS measurement of homocysteine, red: internal standards, blue: samples. *P < 0.05. **c** Schemati**c** diagram showing the significant role of gut microbiota in host hyperhomocysteinemia and glucose intolerance under HM diet. Under the HM diet, the gut microbiota produced and secreted more homocysteine, likely due to the increased abundance of *Faecalibaculum* and *Dubosiella*. Furthermore, gut microbiota-derived homocysteine induced hyperhomocysteinemia and glucose intolerance in mice. Hcy homocysteine, CGS cystathionine gamma-synthase, CCBL cysteine-S-conjugate beta-lyase.

We then investigated the correlation between the changes in the gut microbiota composition and the levels of plasma homocysteine of chow diet-fed mice and HM diet-fed mice. Plasma homocysteine levels were positively correlated with the abundances of the genera *Faecalibaculum* (*P* = 0.0035, *r* = 0.7684) and *Dubosiella* (*P* = 0.0187, *r* = 0.6632; Fig. 6a). We further analyzed the correlation between the changes in the gut microbiota composition and the gene abundance of CGS and CCBL, which were involved in bacterial homocysteine production in the de novo methionine biosynthesis pathway. The abundance of *Faecalibaculum* was positively correlated with the gene abundance of CCBL (*P* = 0.0019, *r* = 0.7972), while the abundance of *Dubosiella* was positively correlated with the gene abundance of CGS (*P* = 0.0168, *r* = 0.6713; Fig. 6a). *Faecalibaculum* and *Dubosiella* genera were identified as putative producers of homocysteine under the HM diet. To detect whether *Dubosiella* could produce homocysteine in vitro, we performed LC‒MS analysis to detect homocysteine from *Dubosiella newyorkensis*-cultured supernatant. We found the same peak retention time (~3.47 min) from the culture supernatant with d,l-homocysteine standards (Fig. 6b). Taken together, *Faecalibaculum* and *Dubosiella* potentially produced homocysteine.

## Discussion

In this study, we revealed the significant role of the gut microbiota in HM diet-induced hyperhomocysteinemia (HHcy) and glucose intolerance. Via the combination of antibiotics to ablate the gut microbiota and metagenomic analysis, we found that *Faecalibaculum* and *Dubosiella* were potential producers of homocysteine (Hcy) under an HM diet. Targeting the gut microbiota may shed light on the prevention and treatment of HHcy-associated diseases.

The major finding of our study is the vital role of the gut microbiota in homocysteine metabolism. It has long been recognized that the liver is a major site of methionine and homocysteine metabolism. However, previous studies found that plasma Hcy levels were notably higher in piglets fed enterally than parenterally, implying that the gastrointestinal tissue (GIT) exports large amounts of Hcy^34^. A recent study using the isotope-labeling method showed that 20% of the dietary methionine intake was metabolized by the GIT^24^. Methionine was metabolized into Hcy (31%), CO_2_ (40%), or tissue protein (29%) in the GIT of piglets, while most of the whole-body methionine intake (80%) was used to synthesize protein, and only 20% was metabolized through transmethylation and transsulfuration^24^. Therefore, the GIT is a vital site of net Hcy production. The gastrointestinal tract harbors numerous microbiota, which exert an important effect on the host during homeostasis and disease. However, the role of the gut microbiota in methionine and homocysteine metabolism remains elusive. In our study, we administered antibiotics to deplete the gut microbiota and revealed the significant role of the gut microbiota in HM diet-induced HHcy. Metagenomic analysis showed that the abundances of two bacterial homocysteine biosynthesis-related KOs were elevated under the HM diet, implying an increased ability of intestinal bacterial homocysteine production. Moreover, homocysteine was detected from *Dubosiella newyorkensis*-cultured supernatant by LC‒MS analysis. Recently, Rosario et al. found that *Akkermansia muciniphila*, *Eubacterium* sp., *Subdoligranulum* sp., and Clostridiales Family XIII bacterium were positively correlated with plasma homocysteine levels in patients with Parkinson’s disease^35^. Herein, we provide direct cause-and-effect evidence of the relationship between the gut microbiota and HM diet-induced hyperhomocysteinemia. In our study, ABX treatment did not affect plasma and intestinal epithelial methionine levels under the HM diet. A probable reason is that gut microbiota metabolized partial methionine, while the vast majority of methionine was metabolized in the liver. So ABX treatment did not affect methionine levels but homocysteine levels as gut microbiota produced a proportion of homocysteine utilizing methionine. ABX treatment reduced intestinal epithelial homocysteine levels under the HM diet, implying intestinal epithelium tissue probably exported less homocysteine produced by gut microbiota.

Another interesting finding of our study is that *Faecalibaculum* and *Dubosiella* potentially produced homocysteine under the HM diet. Diet is a crucial determinant of gut microbiota structure and function. *Faecalibaculum* and *Dubosiella* were enriched in mice fed a high-fat diet^36,37^. Recently, a study showed that *Faecalibaculum rodentium* was positively correlated with phospholipids, indicating the vital role of *Faecalibaculum rodentium* in phospholipid synthesis^38^. Moreover, dietary phosphatidylcholine and sphingomyelin supplementation reversed the decreased abundance of *Faecalibacterium* and *Dubosiella* in the dextran sulfate sodium (DSS)-induced colitis mouse model^39^. Lai et al. found that *Lycium barbarum* polysaccharide (LBP) intervention increased the abundances of *Faecalibacterium* and *Dubosiella*, which may generate S-adenosylmethionine (SAM), a methionine metabolite, in a rheumatoid arthritis rat model^40^. In our study, we proposed that the HM diet increased the abundances of *Faecalibacterium* and *Dubosiella*, which might produce and secrete homocysteine, inducing host hyperhomocysteinemia and glucose intolerance (Fig. 6c). The de novo biosynthetic pathway of methionine is conserved across intestinal bacteria but absent from mammalian hosts^41^. In the de novo methionine biosynthesis pathway, cysteine is transferred to homoserine to form cystathionine, which is cleaved to produce homocysteine. These reactions are catalyzed by the enzymes CGS and CCBL^42^. In our study, we found that the abundance of *Faecalibaculum* was positively correlated with the bacterial gene abundance of CCBL, while the abundance of *Dubosiella* was positively correlated with the bacterial gene abundance of CGS. To explore the role of CGS and CCBL in intestinal microbial homocysteine biosynthesis, we may need to construct mutant bacterial strains of CGS and CCBL in the future.

Our study provides more evidence that the gut microbiota participates in the regulation of glucose homeostasis. Glucose intolerance is characterized by a state of persistent hyperglycemia after a glucose load. Most individuals with impaired glucose tolerance eventually develop diabetes^43^. Under the chow diet, gut microbiota-deficient mice showed accelerated glucose clearance ability due to brown adipose tissue^44^. Previous studies showed that gut microbiota ablation using antibiotic treatment alleviated high-fat diet-induced glucose intolerance^45,46^. A cross-sectional study found that patients with impaired glucose tolerance have higher plasma homocysteine levels (OR = 1.508, *P* = 0.007)^47^. It has been reported that elevated plasma homocysteine levels contribute to glucose intolerance in mice^11,12^. In our study, we found that gut microbiota depletion improved glucose tolerance under a high-methionine diet, likely by lowering plasma homocysteine levels.

In summary, we discovered that the gut microbiota produced homocysteine under an HM diet and that antibiotic administration reduced plasma levels of homocysteine. For patients with hyperhomocysteinemia, targeting the gut microbiota might be a potential strategy to reduce plasma homocysteine levels. However, the current study lacked clinical evidence of gut microbiota structural and functional changes in patients with hyperhomocysteinemia. Therefore, further relevant studies are still necessary in the future.

## Methods

### Mice

Male C57BL/6J mice (8 weeks old) were fed a standard diet or a high-methionine (2%, 20 g/kg l-methionine) diet (HFK Biosciences, Beijing, China) for 4 weeks to establish the animal model of hyperhomocysteinemia^19^. All mice were raised under a 12-h light/12-h dark cycle with free access to food and water. Mice were housed in groups of 3 mice per cage during the experiment. The daily consumption of food was recorded every day before the food administration. All mice were intraperitoneally anesthetized with 1.25% avertin at the dose of 15 μL/g body weight before being sacrificed. All animal studies were performed in compliance with the guidelines of the Institute of Laboratory Animal Resources and were approved by the Animal Care and Use Committee of Peking University.

### Antibiotic treatment

Mice were treated by adding an antibiotic solution (ABX) containing neomycin (1 mg/mL, final concentration in drinking water), streptomycin (1 mg/mL, final concentration in drinking water), and bacitracin (1 mg/mL, final concentration in drinking water) (Sigma-Aldrich) to sterile drinking water for 28 days^48^.

### Mouse fecal sample collection and DNA extraction

Mice were kept in an empty cage without bedding for 15 min to gather fresh stool samples into sterile centrifuges. Tubes were stored at −80 °C until analysis. Fecal DNA was extracted using the TIANamp stool DNA Kit (TIANGEN Biotech, Beijing, China) according to the manufacturer’s instructions. To quantify the total bacterial load, 16S rRNA gene primers based on the dual priming oligonucleotide (DPO) principle (16SDPO-forward 5’-AGAGTTTGATCMTGGCTCA-I-I-I-I-I-AACGCT-3’; 16SDPO-reverse 5’-CGCGGCTGCTGGCAI-I-I-A-I-TTRGC-3’) were used for quantitative real-time PCR assays^49,50^.

### Glucose tolerance test (GTT) and insulin tolerance test (ITT)

For the glucose tolerance test (GTT), mice were fasted for 12 h before the administration of glucose (3 g/kg, i.p.)^51^. For the insulin tolerance test (ITT), mice fasted for 4 h before the administration of insulin (1 IU/kg, i.p.)^51^. Blood samples were drawn from a cut at the tip of the mouse tail at 0, 15, 30, 60, 90, and 120 min, and blood glucose concentrations were detected immediately using a glucose meter (Accu-Chek, Roche, Switzerland). The area under the blood glucose curve (AUC) was used to quantify the differences between GTT and ITT.

### Plasma metabolite measurement

Plasma homocysteine levels were estimated using commercially available enzymatic cycling methods and read with a Hitachi 7600 Biochemistry Automatic Analyzer (Hitachi, Tokyo, Japan). Plasma insulin was examined by an ELISA kit (Dogesce, Beijing, China). Plasma triglyceride (TG) and total cholesterol (TC) were measured by commercial kits from Biosino Biotechnology and Science (Beijing, China). Plasma triglyceride levels were measured by GPO-PAP method. The triglyceride GPO-PAP method is based on the disruption of triglyceride by lipoprotein lipase, which breaks it apart into glycerol and free fatty acids. The assay measures the glycerol component concentration, which is proportional to the triglyceride concentration. Plasma total cholesterol levels were measured by CHOD-PAP method. In the first step, cholesterol esterase hydrolyzes cholesterol esters to yield cholesterol and free fatty acids. In the next step, cholesterol oxidase catalyzes the oxidation of cholesterol. Under the catalytic action of peroxidase, hydrogen peroxide produced in the previous reaction oxidizes the chromophore 4-aminophenazone to the red dye quinone imide. The color intensity of the red dye product is measured photometrically, at 500 nm, and is proportional to total cholesterol concentration.

### Liver enzymatic activity assay

Liver CBS enzymatic activity was determined using a Cystathionine β-Synthase Activity Assay kit (BioVision, San Francisco, USA) following the manufacturer’s instructions. BioVision’s Cystathionine β Synthase Assay kit utilizes cysteine and homocysteine as substrates to produce H_2_S. Hydrogen sulfide reacts with the azido-functional group of the azido-functional group of fluorescent probe yielding a fluorescent amino group (Ex/Em = 368/460 nm).

$${\rm{Cysteine}}\,{+}\,{\rm{Homocysteine}}\,\mathop{\longrightarrow}\limits^{{\rm{CBS}}}{\rm{Cystathionine}}\,{+}\,{{\rm{H}}}_{{2}}{\rm{S}}\,\mathop{\longrightarrow}\limits^{{\rm{CBS}}\,{\rm{Probe}}}{\rm{Fluorescence}}$$

### Liver SAM and SAH levels measurement

Liver SAM level was examined by an ELISA kit (Meimian, Jiangsu, China). Liver SAH level was examined by an ELISA kit (Meimian, Jiangsu, China).

### RNA extraction and quantitative real-time PCR analysis

Total RNA from mouse liver tissues was isolated using TRIzol reagent (Vazyme Biotech Co., Nanjing, China). Total RNA (1 µg) was reverse transcribed into cDNA using a reverse transcription system (Vazyme Biotech Co., Nanjing, China). SYBR Green 2× polymerase chain reaction (PCR) mix (Vazyme Biotech Co., Nanjing, China) was used for quantitative real-time PCR amplification^52^. All samples were normalized to β-actin. The primer sequences for quantitative real-time PCR are as follows: β-actin, 5’-GGCTGTATTCCCCTCCATCG-3’ (sense) and 5’-CCAGTTGGTAACAATGCCATGT-3’ (antisense); CBS, 5’-GGCCTGATACCCCAAGCAG-3’ (sense) and 5’-GGTGTTCCCAATTTTCCTCAGAA-3’ (antisense); BHMT, 5’-AATGCCGGAGAAGTTGTGATT-3’ (sense) and 5’-ACTCCCGATGAAGCTGACGA-3’ (antisense); MetRS, 5’-CTTGAGTCGTCAAAACTGTCCT-3’ (sense) and 5’-GTCTGGAACCAACTTTGCAGG-3’ (antisense); MAT1A, 5’- GTGCTGGATGCTCACCTCAAG-3’ (sense) and 5’-CCACCCGCTGGTAATCAACC-3’ (antisense); MAT2A, 5’-GCTTCCACGAGGCGTTCAT-3’ (sense) and 5’-AGCATCACTGATTTGGTCACAA-3’ (antisense); MTHFR, 5’-GGCAGCGAGAGTTCCAAGG-3’ (sense) and 5’-CAGGGAGAACCACTTGTCACC-3’ (antisense).

### Western blotting

Mouse liver tissue lysates were resolved by 10% SDS–PAGE for western blot analysis and then were transferred onto nitrocellulose membranes. After being blocked with 5% milk in TBST, the blots were incubated with primary antibodies at 4 °C overnight, followed by secondary antibody incubation for 1 h. Primary antibodies were as follows: GAPDH (60004-1-Ig, 1:1000, Proteintech), CBS (14787-1-AP, 1:1000, Proteintech), and BHMT (15965-1-AP, 1:1000, Proteintech). Immunofluorescence images were obtained by Odyssey infrared imaging (LI-COR Biosciences, Lincoln, NE).

### Metagenomic sequencing

DNA concentrations were measured using a Qubit® dsDNA Assay Kit in a Qubit® 2.0 Fluorometer (Life Technologies, CA, USA). A total amount of 1 µg of DNA per sample was used as input material for the DNA sample preparations. Sequencing libraries were generated using the NEBNext® Ultra DNA Library Prep Kit for Illumina (NEB, USA) following the manufacturer’s recommendations, and index codes were added to attribute sequences to each sample. Briefly, the DNA sample was fragmented by sonication to a size of 350 bp, and then DNA fragments were end-polished, A-tailed, and ligated with the full-length adaptor for Illumina sequencing with further PCR amplification. Finally, PCR products were purified (AMPure XP system), and libraries were analyzed for size distribution by an Agilent 2100 Bioanalyzer and quantified using real-time PCR.

### Metagenomic analysis

Preprocessing of the raw data obtained from the Illumina HiSeq sequencing platform using Readfq was conducted to acquire clean data for subsequent analysis. Clean data were blasted to the host database by default using Bowtie2.2.4 software to filter the reads that were of host origin. All the reads not used in the forward step of all samples were combined, and then SOAPdenovo (V2.04)/MEGAHIT (v1.0.4-beta) software was used for mixed assembly with the same parameters as a single assembly. The mixed assembled scaffolds were broken at the N connection, and the scaftigs were obtained. The fragments shorter than 500 bp in all scaftigs generated from single or mixed assemblies were filtered for statistical analysis. DIAMOND software was used to blast the Unigenes to the sequences of Bacteria, Fungi, Archaea, and Viruses, which were all extracted from the NR database of NCBI. For the final aligned results of each sequence, as each sequence may have multiple aligned results, the result for which the *e* value was ≤the smallest *e* value*10 was chosen for the LCA algorithm, which was applied to the system classification using MEGAN software to ensure the species annotation information of sequences. The table containing the number of genes and the abundance information of each sample in each taxonomy hierarchy (kingdom, phylum, class, order, family, genus, and species) was obtained based on the LCA annotation result and the gene abundance table. The abundance of a species in one sample equals the sum of the gene abundance annotated for the species; the gene number of a species in a sample equals the number of genes whose abundance is nonzero. The Krona analysis, relative abundances, cluster heatmap, PCA, and NMDS decrease-dimension analysis were based on the abundance table of each taxonomic hierarchy. The difference between groups was tested by ANOSIM. Metastats and LEfSe analysis were used to identify the different species between groups. A permutation test between groups was used in Metastats analysis for each taxonomy, and the *P* value was obtained. Then, the Benjamini and Hochberg false discovery rate was used to correct the *P* value and acquire the *q* value. LEfSe analysis was conducted by LEfSe software. DIAMOND software (V0.9.9) was used to blast unigenes against the functional database. For each sequence’s blast result, the best Blast hit was used for subsequent analysis.

### Bacterial strain and culture

*Dubosiella newyorkensis* was purchased from ATCC. *Dubosiella newyorkensis* was cultured in 50 mL of chopped meat carbohydrate (CMC) broth at 37 °C in an anaerobic chamber for 24 h. The bacterial culture supernatant was obtained by centrifugation at 8000×*g* for 10 min at 4 °C.

### Liquid chromatography–tandem mass spectrometry (LC‒MS) measurement of homocysteine and methionine

Intestines were dissected free of mesentery from the duodenum to the ileum and cut along the midline. The intestines were placed with the inner face upside, and the epithelia were directly collected by a cell scraper. Then the collected intestinal epithelia were homogenated with an appropriate amount of phosphate-buffered saline (PBS, 5 mL/g tissue) in the glass tube homogenizer. The intestinal epithelial supernatants were collected after centrifugation at 13,000×*g* for 10 min at 4 °C and then were used for LC‒MS measurement.

Plasma methionine, intestinal epithelial methionine, intestinal epithelial homocysteine and culture supernatant homocysteine were measured by LC‒MS. Standards of d,l-homocysteine and l-methionine were purchased from Sigma-Aldrich (USA). The isotopically labeled compounds used as internal standards were d,l-homocysteine-d_4_ from Toronto Research Chemicals (Canada), and H_3_-l-methionine from ISOREAG (Shanghai, China). The reagents for standards and sample preparation, including acetonitrile (ACN) and 1,4-dithiothreitol (DTT), were purchased from Merck (Germany). Formic acid (FA) was purchased from Sigma-Aldrich (USA). In the experiment, 20 µL of supernatant was used, and 20 μL of water, 20 μL of isotopically labeled compounds, and 40 μL of DTT (500 mM) were also added. The solution was mixed and stored at room temperature for 15 min. Then, 300 μL of ACN (0.1% FA) was added. The mixture was centrifuged for 20 min at 14,000 rpm. For LC‒MS analysis, 100 μL of supernatant was used. LC‒MS was performed on an AB Sciex TripleTOF 5600TM mass spectrometer system (AB SCIEX, USA)^53^. Homocysteine and methionine were identified by comparing sample peak retention times with standards, respectively. Concentrations were determined by an external standard calibration method.

### Statistical analysis

Data are presented as the mean ± SEM and were analyzed using GraphPad Prism software. The Shapiro–Wilk normality test was used to determine whether the data were normally distributed. For normal distribution comparisons, unpaired two-tailed Student’s *t*-test or unpaired one-way ANOVA with Tukey’s post hoc analysis was performed. The Mann‒Whitney test was used to compare non-normally distributed data. Data were expressed as the means ± SEM. *P* < 0.05 was considered statistically significant.

## Supplementary information

Supplementary information

Reporting-summary

## Data Availability

Metagenomic sequencing data obtained in the study were deposited in the NCBI Sequence Read Archive under accession number PRJNA892228.
