# Supplementary material for: Ablation of the gut microbiota alleviates high-methionine diet-induced hyperhomocysteinemia and glucose intolerance in mice
Source: NPJ Sci Food. 2023 Jul 17;7:36. doi: 10.1038/s41538-023-00212-3 (PMC10352305; doi:10.1038/s41538-023-00212-3)
Supplement: Supplementary file 1 — Supplementary information [file 41538_2023_212_MOESM1_ESM.pdf]

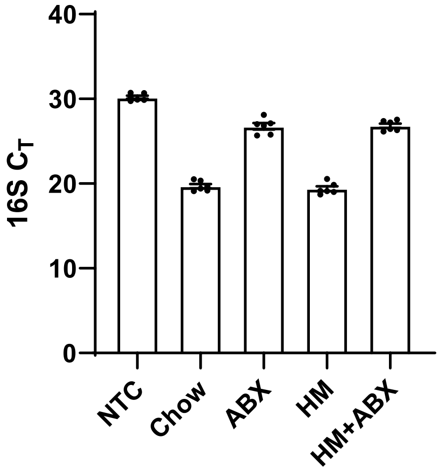

Supplementary Figure 1. qPCR analysis of gut microbial abundance. C_T, Cycle Threshold. NTC: no template control. The values are expressed as the means ± SEM, n = 6 per group. Significance was determined by unpaired one-way ANOVA with Tukey's post hoc test. **P* < 0.05.

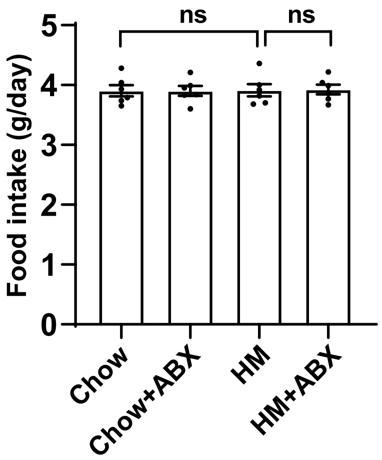

Supplementary Figure 2. Food intake of mice. The values are expressed as the means ± SEM, n = 6 per group. Significance was determined by unpaired one-way ANOVA with Tukey's post hoc test. *P < 0.05.

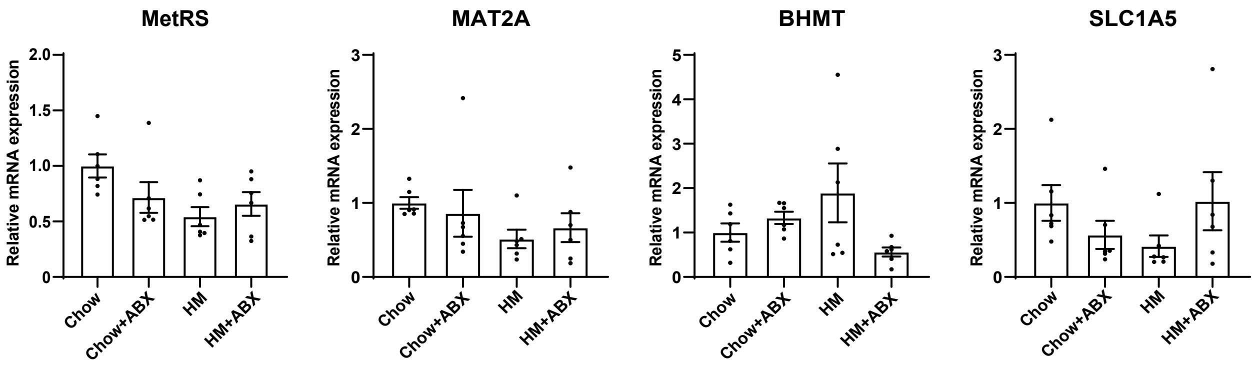

Supplementary Figure 3. mRNA levels of MetRS, MAT2A, BHMT and SLC1A5 in the intestinal epithelium of the four groups. The values are expressed as the means ± SEM, n = 6 per group. Significance was determined by unpaired one-way ANOVA with Tukey's post hoc test.

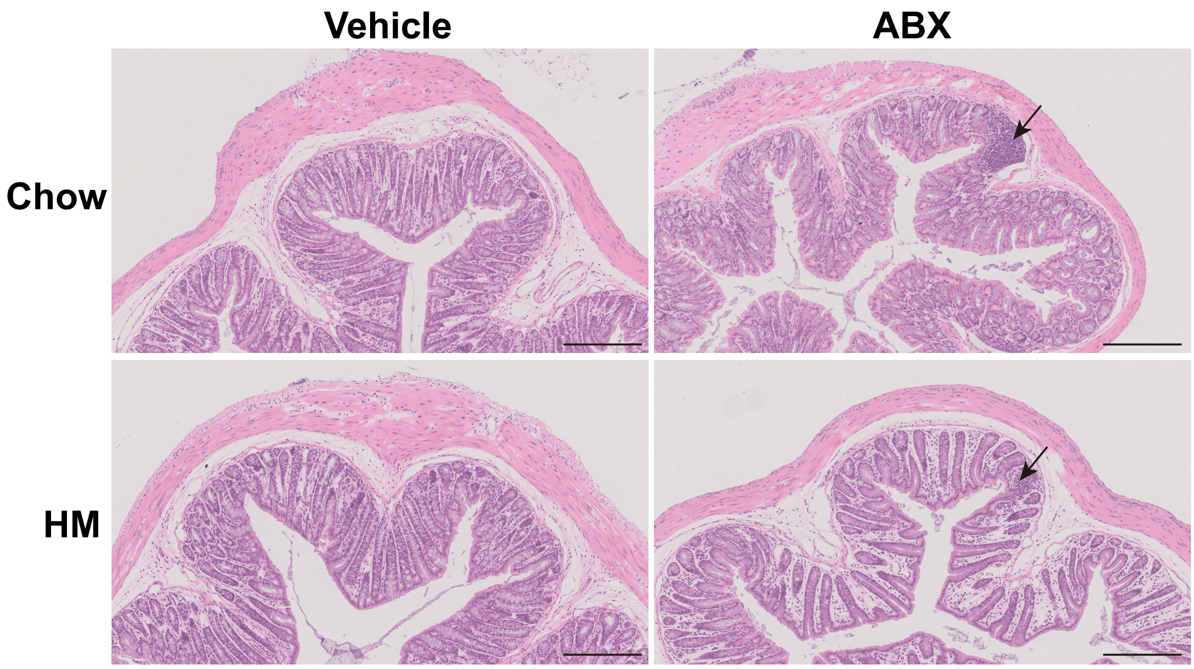

Supplementary Figure 4. H&E-stained colon sections. Black arrows represent lymphocyte infiltration. Scale bar = 250 μm.

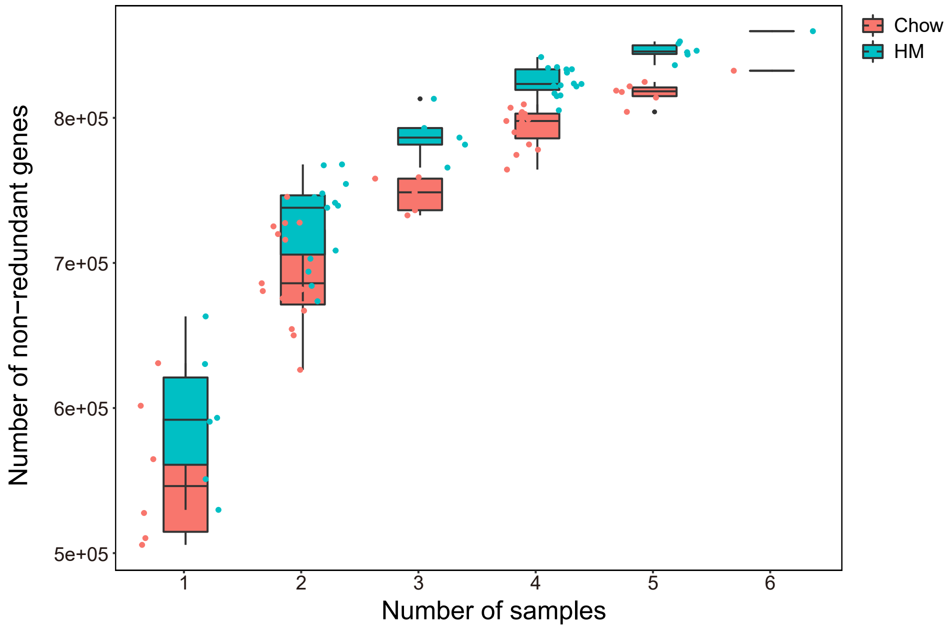

Supplementary Figure 5. Rarefaction curve of each sample in the chow and HM groups. For box plots, the midline represents the median, the box represents the interquartile range (IQR) between the first and third quartiles, and the whiskers represent the lowest or highest values within 1.5 times the IQR from the first or third quartiles.
